# Supplementary material for: Mycofabrication of Mycelium-Based Leather from Brown-Rot Fungi
Source: J Fungi (Basel). 2022 Mar 19;8(3):317. doi: 10.3390/jof8030317 (PMC8950489; doi:10.3390/jof8030317)
Supplement: Supplementary file 1 [file jof-08-00317-s001.zip › jof-1625053-supplementary v2/Supplementary_Figs.pptx]

## Slide 1
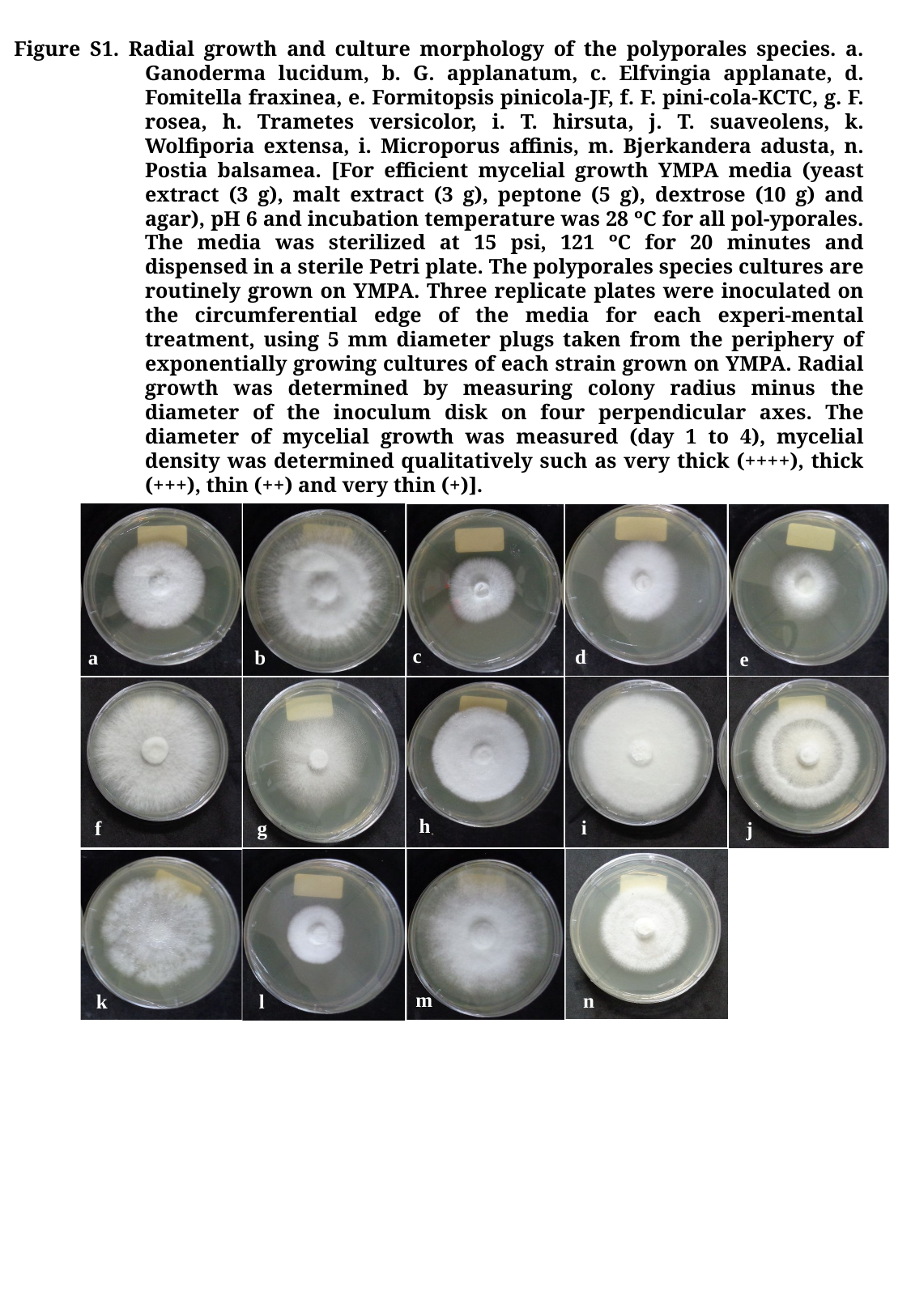

Figure S1. Radial growth and culture morphology of the polyporales species. a. Ganoderma lucidum, b. G. applanatum, c. Elfvingia applanate, d. Fomitella fraxinea, e. Formitopsis pinicola-JF, f. F. pini-cola-KCTC, g. F. rosea, h. Trametes versicolor, i. T. hirsuta, j. T. suaveolens, k. Wolfiporia extensa, i. Microporus affinis, m. Bjerkandera adusta, n. Postia balsamea. [For efficient mycelial growth YMPA media (yeast extract (3 g), malt extract (3 g), peptone (5 g), dextrose (10 g) and agar), pH 6 and incubation temperature was 28 ºC for all pol-yporales. The media was sterilized at 15 psi, 121 ºC for 20 minutes and dispensed in a sterile Petri plate. The polyporales species cultures are routinely grown on YMPA. Three replicate plates were inoculated on the circumferential edge of the media for each experi-mental treatment, using 5 mm diameter plugs taken from the periphery of exponentially growing cultures of each strain grown on YMPA. Radial growth was determined by measuring colony radius minus the diameter of the inoculum disk on four perpendicular axes. The diameter of mycelial growth was measured (day 1 to 4), mycelial density was determined qualitatively such as very thick (++++), thick (+++), thin (++) and very thin (+)].
c
d
a
b
e
h
i
f
g
j
m
n
k
l

## Slide 2
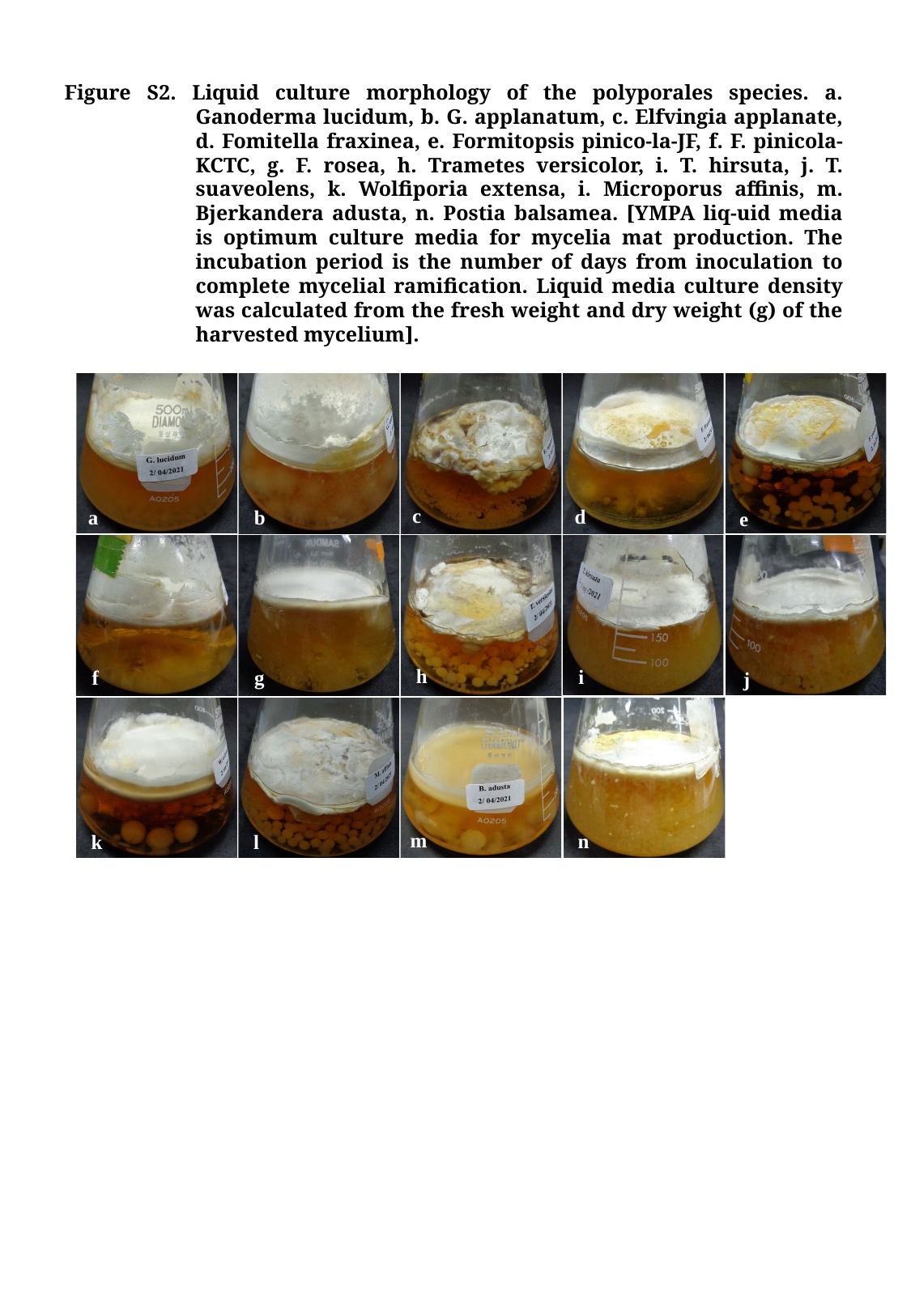

Figure S2. Liquid culture morphology of the polyporales species. a. Ganoderma lucidum, b. G. applanatum, c. Elfvingia applanate, d. Fomitella fraxinea, e. Formitopsis pinico-la-JF, f. F. pinicola-KCTC, g. F. rosea, h. Trametes versicolor, i. T. hirsuta, j. T. suaveolens, k. Wolfiporia extensa, i. Microporus affinis, m. Bjerkandera adusta, n. Postia balsamea. [YMPA liq-uid media is optimum culture media for mycelia mat production. The incubation period is the number of days from inoculation to complete mycelial ramification. Liquid media culture density was calculated from the fresh weight and dry weight (g) of the harvested mycelium].
c
d
a
b
e
h
i
f
g
j
m
n
k
l

## Slide 3
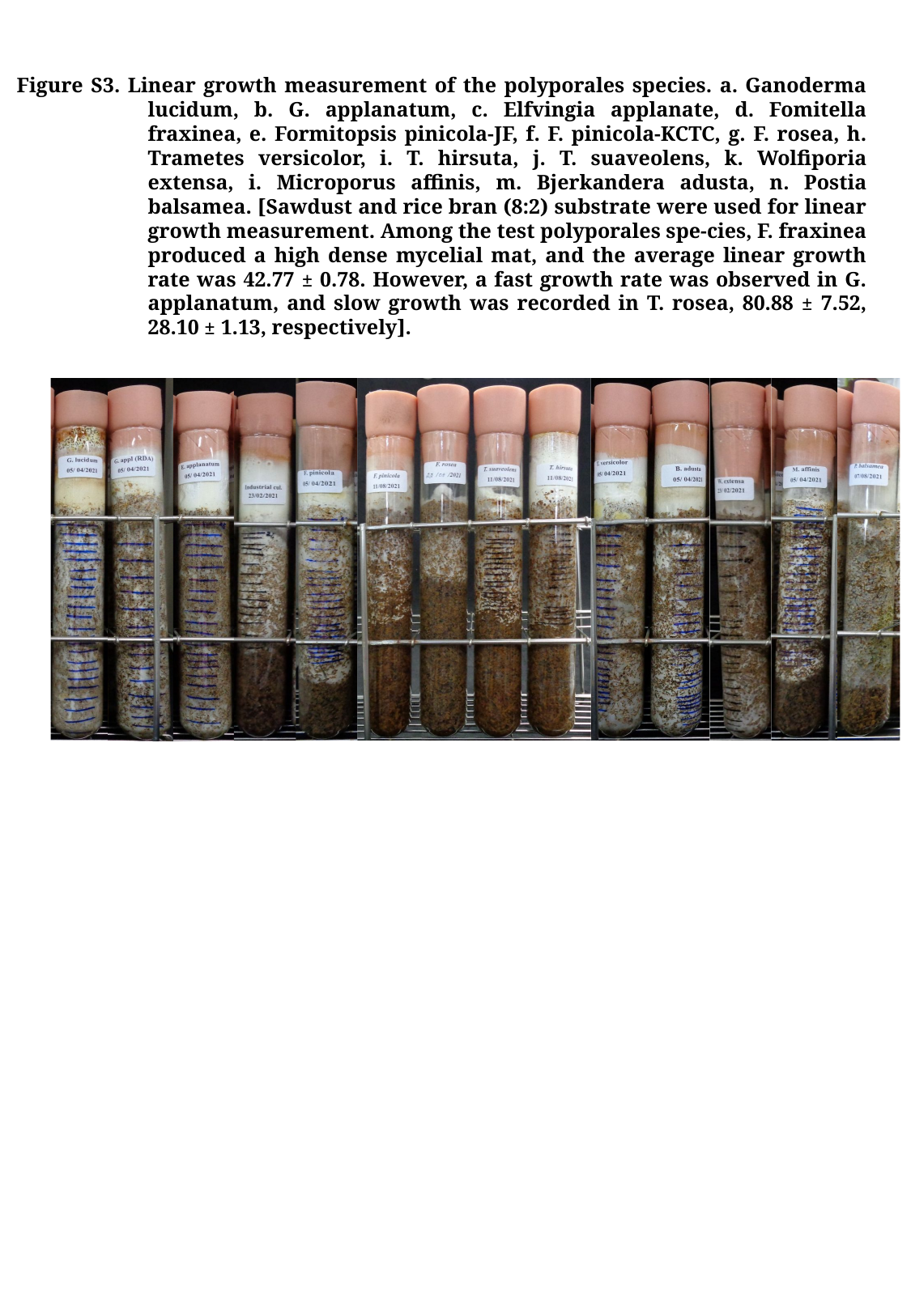

Figure S3. Linear growth measurement of the polyporales species. a. Ganoderma lucidum, b. G. applanatum, c. Elfvingia applanate, d. Fomitella fraxinea, e. Formitopsis pinicola-JF, f. F. pinicola-KCTC, g. F. rosea, h. Trametes versicolor, i. T. hirsuta, j. T. suaveolens, k. Wolfiporia extensa, i. Microporus affinis, m. Bjerkandera adusta, n. Postia balsamea. [Sawdust and rice bran (8:2) substrate were used for linear growth measurement. Among the test polyporales spe-cies, F. fraxinea produced a high dense mycelial mat, and the average linear growth rate was 42.77 ± 0.78. However, a fast growth rate was observed in G. applanatum, and slow growth was recorded in T. rosea, 80.88 ± 7.52, 28.10 ± 1.13, respectively].

## Slide 4
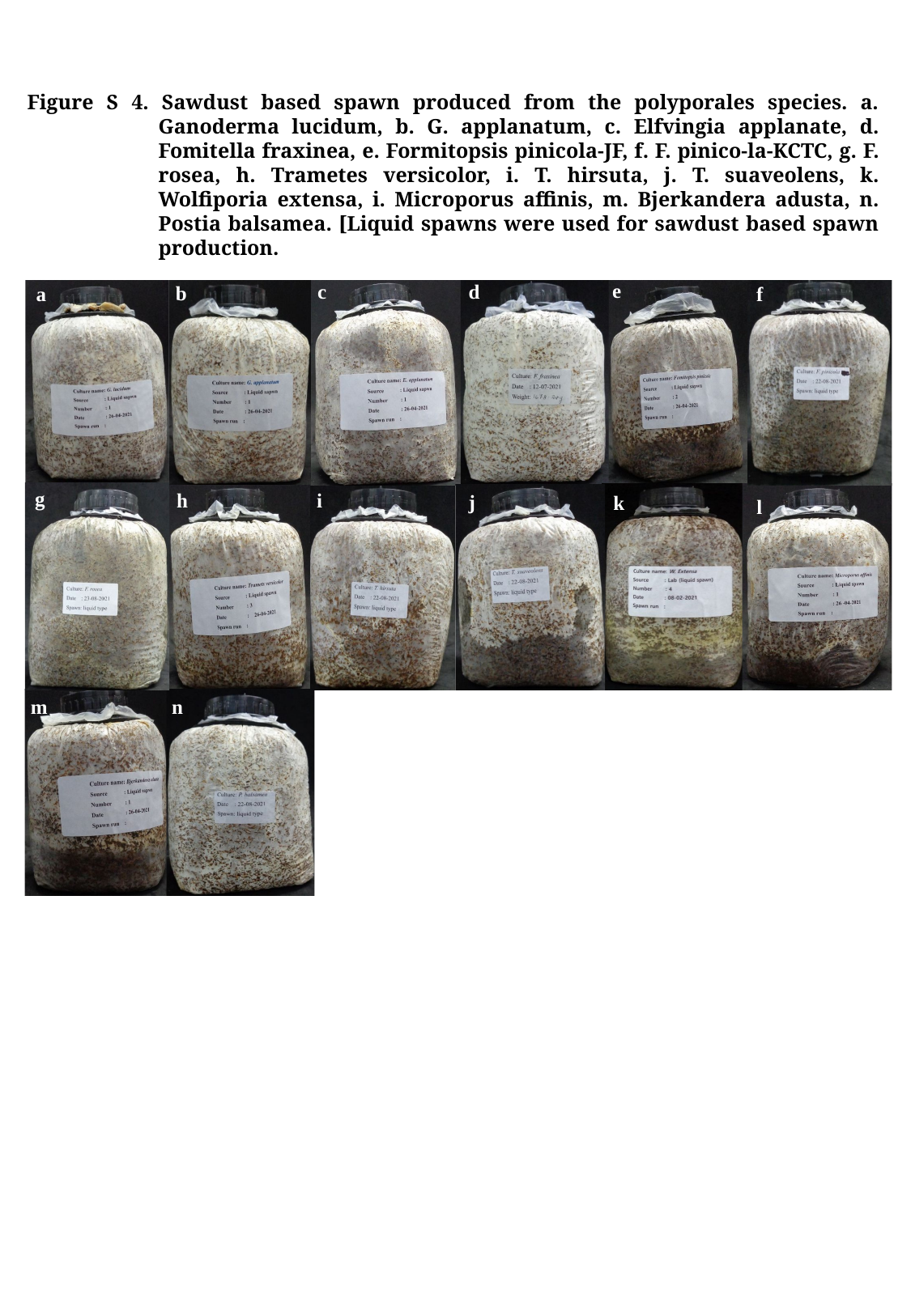

Figure S 4. Sawdust based spawn produced from the polyporales species. a. Ganoderma lucidum, b. G. applanatum, c. Elfvingia applanate, d. Fomitella fraxinea, e. Formitopsis pinicola-JF, f. F. pinico-la-KCTC, g. F. rosea, h. Trametes versicolor, i. T. hirsuta, j. T. suaveolens, k. Wolfiporia extensa, i. Microporus affinis, m. Bjerkandera adusta, n. Postia balsamea. [Liquid spawns were used for sawdust based spawn production.
e
d
c
b
f
a
j
f
g
h
i
j
k
l
g
m
n

## Slide 5
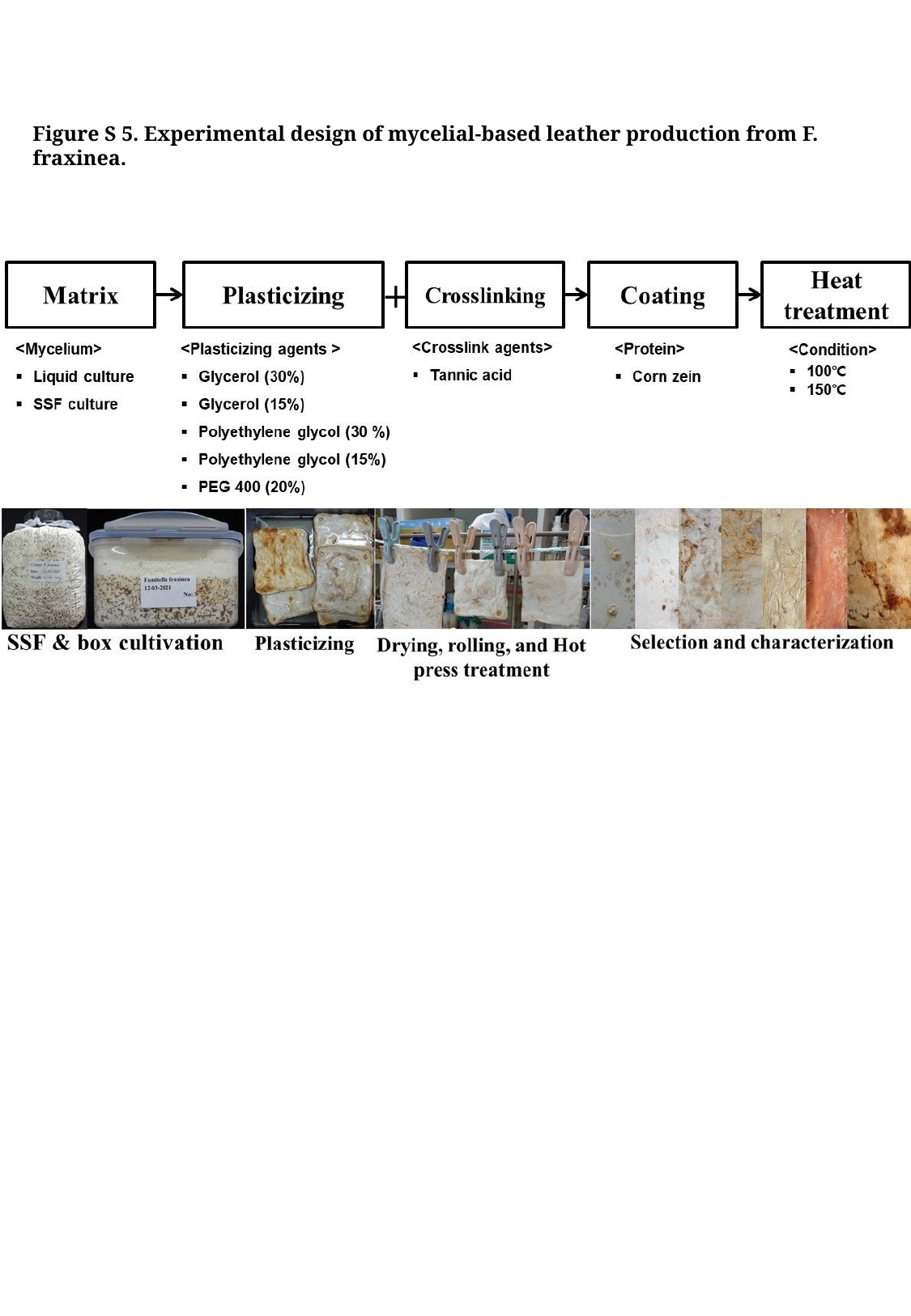

Figure S 5. Experimental design of mycelial-based leather production from F. fraxinea.

## Slide 6
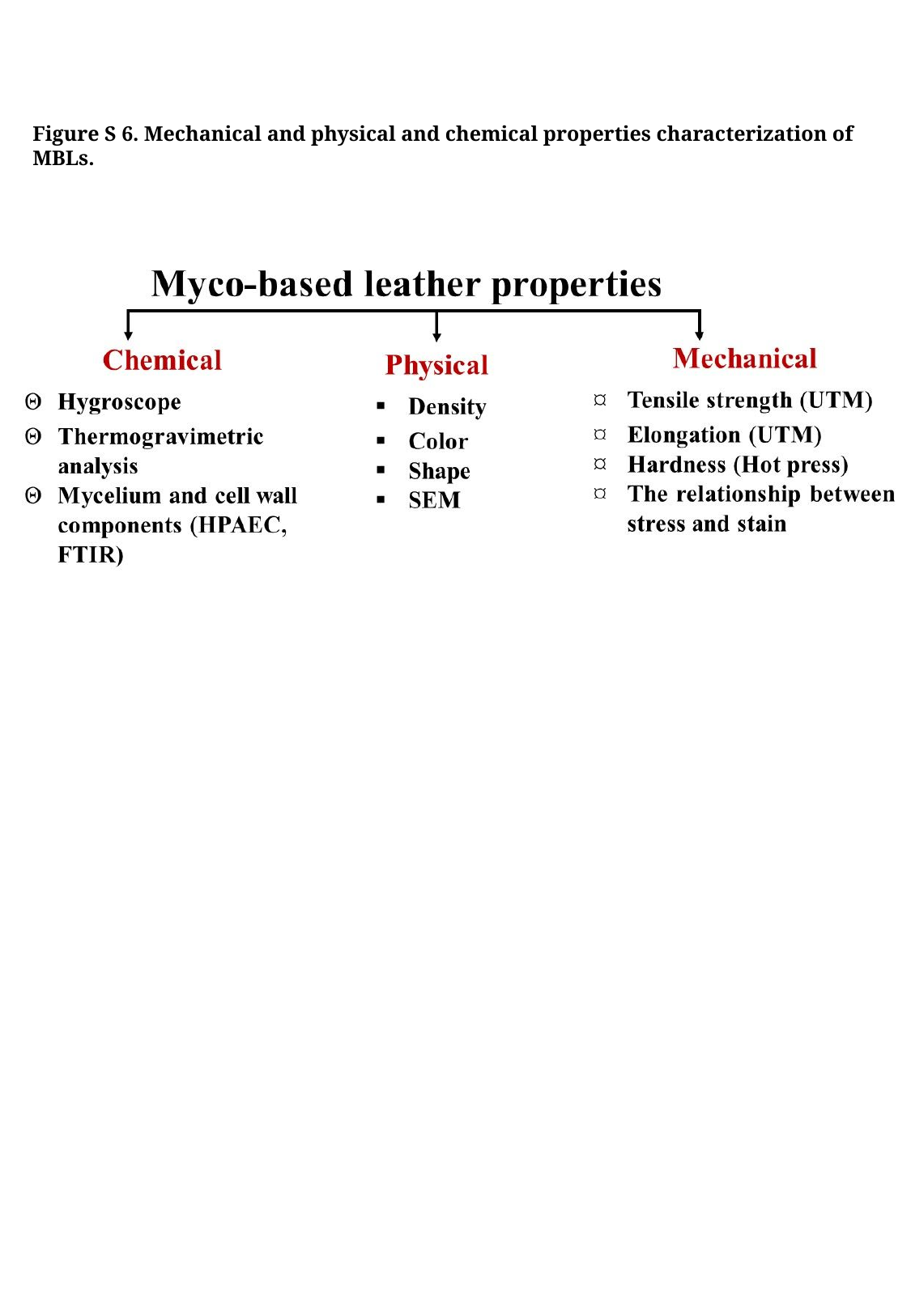

Figure S 6. Mechanical and physical and chemical properties characterization of MBLs.

## Slide 7
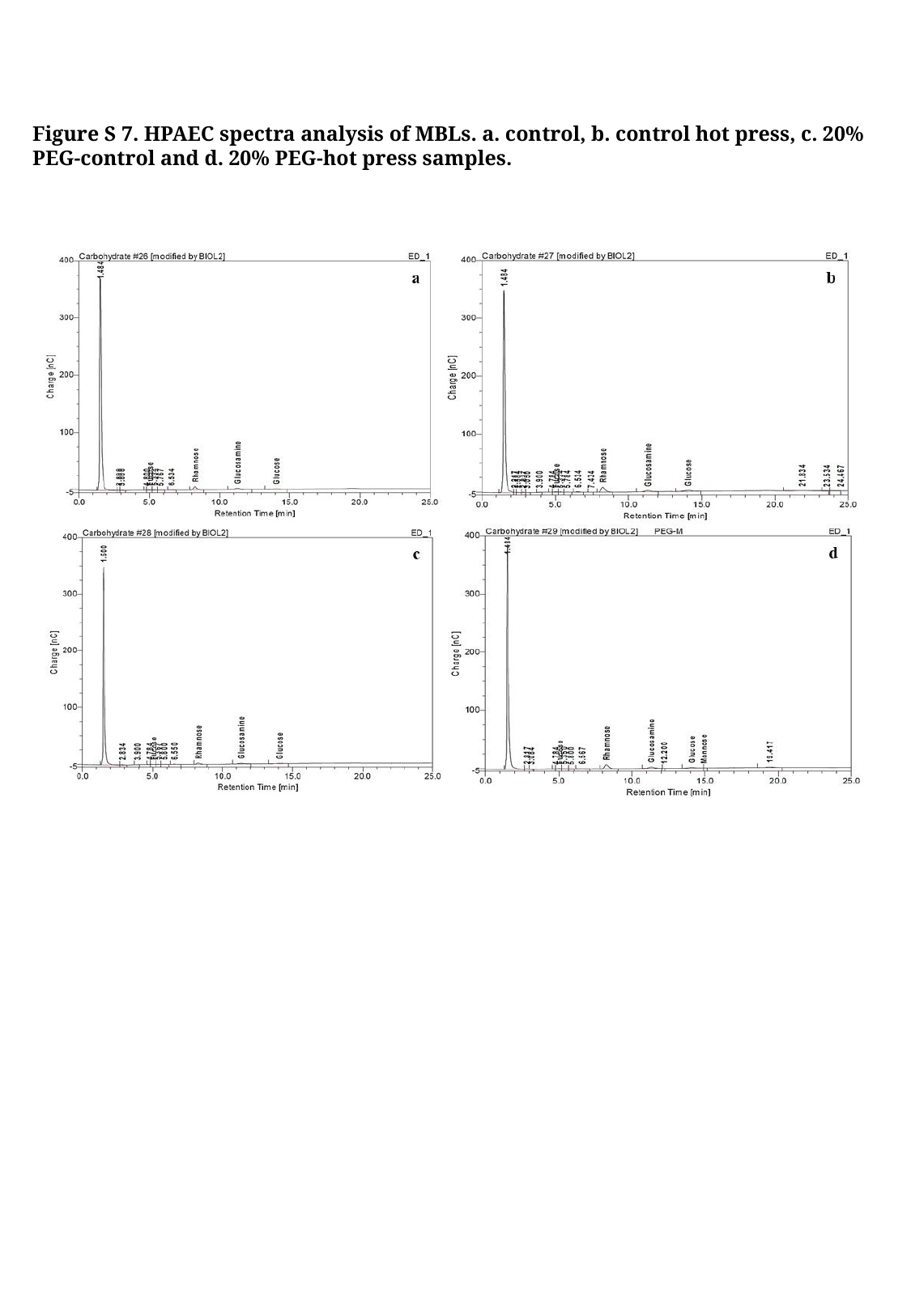

Figure S 7. HPAEC spectra analysis of MBLs. a. control, b. control hot press, c. 20% PEG-control and d. 20% PEG-hot press samples.
