# Supplementary material for: Mycofabrication of Mycelium-Based Leather from Brown-Rot Fungi
Source: J Fungi (Basel). 2022 Mar 19;8(3):317. doi: 10.3390/jof8030317 (PMC8950489; doi:10.3390/jof8030317)
Supplement: Supplementary file 1 [file jof-08-00317-s001.zip › jof-1625053-supplementary v2/Tables.pdf]

Table S1. The density control and MBLs analyzed by Gas Pycnometer (Accupyc Ⅱ 1340/ Micromeritics).

Name	Mass(g)	Volume (cm3)	Density (g/cm3)	Standard Deviation (g/cm3)	Temp. (℃)
PEG-H	2.33	1.60	1.46	0.0007	26.5
PEG-C	1.64	1.21	1.35	0.0011	28.1
C	1.75	1.11	1.58	0.0021	29.8
C H	2.92	1.94	1.51	0.0005	30
